# 

**Published:** 1898-07-09

**Authors:** 


					THE HOSPITAL. "The standard of highest purity."?Lancet, July,9th, 189S.
CADBURY'S "OF fe* CADBURY'S
coooa JFtII lm cocoa
ABSOLUTELY PURE. Jp*i ^ __J NO DRUGS OR ALKALIES USED. ]
No. 615. Vol. XXIV. [H?5*?] Saturday,
July 9th, 1898.
[-SnhBoriptionTerms.] pH(ICE TWOPENCE.

				

